# Supplementary material for: SlMYC2 Mediates the JA Pathway by Responding to Chlorocholine Chloride in the Regulation of Resistance to TYLCD
Source: Plants (Basel). 2025 Apr 30;14(9):1353. doi: 10.3390/plants14091353 (PMC12073760; doi:10.3390/plants14091353)
Supplement: Supplementary file 1 [file plants-14-01353-s001.zip › plants-3550137-supplementary.pdf]

Supplementary Table S1 Sequence information for all primers used in this study

Primer name	Primer sequences	Primer use
qTy-F	GAAACGACCAGTCTGAGGCTGTA	Virus copy number detection
qTy-R	AAGAAACCAATAAGGCGTAAGCGTGTAG	
SlActin-F	TGACGAAGTCAGGACAGGAA	qPCR
SlActin-R	CTGCATCTTCTTTGCCACTG	
MYC2-T1-F	TATTCCTTCAGCTAACGGCGGTTTTAGAGCTAGAAAT	Construction of CRISPR/Cas9 Vectors
MYC2-T1-R	CGCCGTTAGCTGAAGGAATATGACCAATGGTGCTTTG	
MYC2-T2-F	GCAGCCGGAGAGCGACCCATGTTTTAGAGCTAGAAAT	
MYC2-T2-R	ATGGGTCGCTCTCCGGCTGCTGACCAATGTTGCTCC	
Cas9-MYC2-F	CCGCCAGGTTTTGTCCACT	Detection of gene-edited seedlings
Cas9-MYC2-R	TCCACGTCTCTCTAGCACCA	
OE-MYC2-F	acgaacgatagccatggtaccATGACTGAATACAGCTTGCCCAC	Overexpression and its subcellular localization
OE-MYC2-R	gcctgcggccgcgccggatccGTGTGTTTCAGCAATTTTCGATG	
EGFP-F	CTATTGCAGCAATTTAAATCATTT	Overexpression of the seedling assay
EGFP-R	CCGGCGCTCAGTTGGAATTCTAGA	
q-MYC2-F	CTTGTTTACAGGTCAGTCCC	qPCR
q-MYC2-R	CATTCCTTCTTCATTGCTTC	
q-JAZ2-F	AACAATCCACAAAAAAGACA	
q-JAZ2-R	TGGTAAATCAGCAACAGAAG	
ERF5-nLUC-F	acgggggacgagctcggtaccATGGGTTCTCCACAAGAGACTTGT	LCI
ERF5-nLUC-R	gccgggccctctagaggatccTTATATCATAACAAGCTGAGATAATGGTG	
MYC2-cLuc-F	tacgcgtcccggggcggtaccATGACTGAATACAGCTTGCCCAC	
MYC2-cLuc-R	gccgggccctctagaggatccTTAGTGTGTTTCAGCAATTTTCGAT	
JAZ2-NLuc-F	cgagctcggtacccgggatccaATGGGGTCATCGGAAAATATGG	
JAZ2-NLuc-R	cgcgtacgagatctggtcgacGAAATATTGCTCAGTTTTAACAAATTG	
ERF4-nLUC-F	acgggggacgagctcggtaccATGACGAAACAAGATGAAGGATTAAC	
ERF4-nLUC-R	gccgggccctctagaggatccCTACACCAACTCCATCTTGTTCTCTC	
JAZ2-BD-EcoRI-F	atggccatggaggccgaattcAATGGGGTCATCGGAAAATATGG	Y2H
JAZ2-BD-BamHI-R	ccgctgcaggtcgacggatccCTAGAAATATTGCTCAGTTTTAACAAATTG	
ERF4-BD-EcoRI-F	atggccatggaggccgaattcATGACGAAACAAGATGAAGGATTAAC	
ERF4-BD-BamHI-R	ccgctgcaggtcgacggatccCTACACCAACTCCATCTTGTTCTCTC	
ERF5-BD-EcoRI-F	atggccatggaggccgaattcATGGGTTCTCCACAAGAGACTTGT	
ERF5-BD-BamHI-R	ccgctgcaggtcgacggatccTTATATCATAACAAGCTGAGATAATGGTG	
MYC2-AD-EcoRI-F	gccatggaggccagtgaattcATGACTGAATACAGCTTGCCCAC	
MYC2-AD-BamHI-R	cagctcgagctcgatggatccTTAGTGTGTTTCAGCAATTTTCGAT	
